# The safety of introducing a new generation TAVR device: one departments experience from introducing a second generation repositionable TAVR

**DOI:** 10.1186/s12872-016-0466-1

**Published:** 2017-01-13

**Authors:** Henrik Bjursten, Shahab Nozohoor, Malin Johansson, Igor Zindovic, Carl-Fredrik Appel, Johan Sjögren, Magnus Dencker, Göran Olivecrona, Jan Harnek, Sasha Koul, Ted Feldman, Michael J. Reardon, Matthias Götberg

**Affiliations:** 1 Department of Cardiothoracic Surgery, and Cardiology, Institute of Clinical Sciences, Lund University, Skane University Hospital, SE-221 85 Lund, Sweden; 2 Department of Clinical Physiology, and Cardiology, Institute of Clinical Sciences, Lund University, Skane University Hospital, Lund, Sweden; 3 Cardiology Division, Evanston Hospital, Evanston, IL USA; 4 Houston Methodist DeBakey Heart and Vascular Center, Houston, TX USA

## Abstract

**Background:**

In the evolving field of transcatheter aortic valve replacements a new generation of valves have been introduced to clinical practice. With the complexity of the TAVR procedure and the unique aspects of each TAVR device, there is a perceived risk that changing or adding a new valve in a department could lead to a worse outcome for patients, especially during the learning phase. The objective was to study the safety aspect of introducing a second generation repositionable transcatheter valve (Boston Scientific Lotus valve besides Edwards Sapien valve) in a department.

**Methods:**

In a retrospective study, 53 patients receiving the Lotus system, and 47 patients receiving the Sapien system over a period of three years were compared for short-term outcome according to VARC-2 definitions and 1-year survival.

**Results:**

Outcome in terms VARC-2 criteria for early safety and clinical efficacy, stroke rate, and survival at 30 days and at 1 year were similar. The Lotus valve had less paravalvular leakage, where 90% had none or trace aortic insufficiency as compared to only 48% for the Sapien system.

**Conclusions:**

Introduction of a new generation valve can be done with early device success and safety, and without jeopardizing the outcome for patients up to one year. We found no adverse effects by changing valve type and observed improved outcome in terms of lower PVL-rates. Both existing and new centers starting a TAVR program can benefit from the use of a new generation device.

## Background

Transcatheter Aortic Valve Replacements (TAVR) has grown rapidly in the last years, and the outcome in terms of survival is good for a high-risk group of patients [1, 2]. Presently, the self-expanding and balloon expandable systems have dominated, and large randomized clinical studies have established their safety and efficacy [1, 3]. Despite good clinical outcomes these valves exhibit some inherent technical limitations. Specifically paravalvular leak (PVL) and malposition of the valve are two problems that have been associated with adverse outcome [4–6]. Newer valves have been designed to address some of these issues, and recently Boston Scientific introduced a second-generation TAVR device which addressed both PVL and malposition, as it has an adaptive seal and is also fully repositionable and retrievable [7, 8].

With increasing number of interventions and new devices on the market, many centers will need to decide if they should change to a new device, use several devices in their practice or keep using the single device they have largest experience with. Given the complexity of the procedures and the frailty of the patients being treated, this decision to change is not always easy. Using an established device with extensive implantation experience does not subject the patients to a learning curve for the team. On the other hand introducing a second device with different characteristics could increase the ability to individualize therapy for the patient in order to improve outcome.

The Boston Scientific Lotus system was introduced in 2013 in our department. This provided us with an opportunity to study the safety of introducing a new valve in terms of both short-term safety and survival. To our knowledge, there are no randomized studies comparing a TAVR-valve against another, and therefore a retrospective study will provide us with indicative information on performance. The aim of this retrospective study was thus to compare the outcome of the two valves during a well-defined time period in terms of device success, early safety as defined by VARC-2, and 1-year survival.

## Methods

### Study design

Our TAVR-program started in 2007, and since its inception we have used the available balloon-expandable Sapien™ valve system (THV, XT, S3, Edwards Lifescience, Irvine, CA, USA). In 2013 we participated in the REPRISE II study [7, 9] and the repositionable Boston Lotus™ valve system (Boston Scientific, Marlborough, MA) was introduced in the department. After gaining initial clinical experience we decided to use the Boston Lotus as the primary valve when anatomically feasible. This retrospective study includes all transfemoral TAVR performed from 1st of January 2012 to 31st of December 2014 at Skane University Hospital, Lund, Sweden. The study was approved by the local ethics committee (LU 2009/87).

### Patient selection

Patients included all had a severe symptomatic aortic stenosis. They were selected for TAVR either because they were denied conventional surgery, were frail, old age (>85 years) or other clinical reasons that they would benefit from a TAVR by a multidisciplinary team consisting of at least one cardiologist and cardiac surgeon. By performing a TAVR, the patients were expected to have a survival of at least one year, and an increase in quality of life. Only patients suited for a transfemoral access were included in this study, and the other patients were treated with an alternative access by the same team that performed the transfemoral cases.

### Implantation technique

All procedures were performed in a dedicated hybrid operation room with at least one interventional cardiologist and one cardiac surgeon performing the procedure together. In the beginning of the study period, all patients underwent general anesthesia, and during the study period conscious sedation was introduced. For both devices, femoral access was obtained by cut-down or by percutaneous puncture with subsequent closing of the puncture site with a closure device. Heparin was given to achieve an ACT-level > 250 s. Pre-dilatation was performed in the majority of cases, unless the pre-operative CT had a low calcium burden in the native valve. Ventricular rapid pacing was used for all Edwards Sapien valve implantations, and for the balloon valvuloplasty in about one third of the Lotus implantations. The implantation technique for Edwards Sapien and Boston Lotus system have been described in detail elsewhere [1, 7]. Sizing of the valve was performed according to the manufacturers’ recommendations.

### Data sources

Peri-operative data for the study was retrieved from three principal sources. Base-line characteristics and intra-procedural data was obtained from SWEDEHEART, which is a national quality registry including all invasive cardiac procedures in Sweden. This registry also contains survival data for patients. In the case of missing data, additional data were retrieved from the electronic medical records. Early safety according to VARC-2-criteria was registered by retrospectively reviewing the electronic medical records for the patients [10]. Pre- and post-operative echocardiographical examinations were re-assessed by an independent, blinded, and experienced echocardiographer (MD). Post-operative echocardiography was performed the days after implantation.

### Statistics

Continuous data were presented as mean ± one standard deviation and proportions as percent and number. A two-tailed *t*-test was performed for comparison of continuous variables, and a two-tailed Fishers exact test for dichotomous variables. The Kaplan-Meier estimate was used to illustrate survival after valve implantation. Calculations and graphs were made with Statistica version version 12 (Statsoft, Tulsa, OK) and Stata version 14 (Statacorp, Collage station, TX).

## Results

**Fig. 1:**
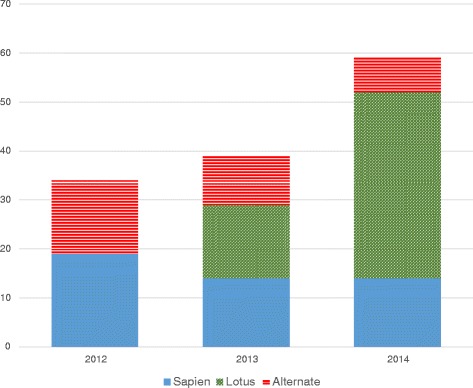
Distribution between Edwards Sapien valve (*blue solid*), Boston Lotus valve (*Green shaded*) and alternate access during the study period (*red striped*) during the study period for transfemoral approach

A total of 100 patients were included in this study, where 47 received the Edwards Sapien valve (40 Sapien XT and 7 Sapien-3), and 53 received the Boston Lotus valve. Size distribution between the valves were similar, but Sapien spanned over a wider range (Table 1). One patient had a device failure with the Lotus system, and returned 42 days later for a successful new Lotus implantation from the contralateral side. Thus, there were 54 Boston Lotus implantations during the study period (implantations are presented with *n* = 54 and patients *n* = 53 for the Lotus in this report). There were more Sapien implants in the beginning of the study period, and more Lotus implants in the end of the study period (Fig. 1).

**Table 1:** Distribution of valves

Size (mm)	Lotus	Sapien-3	Sapien-XT
20			1
23	21	2	13
25	9		
26			15
27	23		
29		5	11
Total	53	7	40

Distribution of valves by type/manufacturer and valve sizes

The patient cohorts differed, where patients in the Lotus group were older (84.1 ± 4.9 vs 77.1 ± 11.5 years, *p* < 0.001), had higher EuroScore I (25.3 ± 12.3 vs 18.2 ± 10.8, *p* < 0.005) and EuroScore II (7.8 ± 4.6 vs. 5.8 ± 4.8, *p* < 0.05, Table 2). The Lotus group had higher pre-operative peak gradient (80.0 ± 17.9 vs 71.7 ± 18.8 mmHg, *p* < 0.05).

**Table 2:** Patient characteristics

	Lotus (*n* = 53)	Sapien (*n* = 47)	*p*-value
Age (years)	84.1 (4.9)	77.1 (11.5)	0.0001
Male	35.8% (19)	53.2% (25)	0.1068
Diabetes mellitus	15.1% (8)	25.5% (12)	0.2186
COPD	7.5% (4)	17% (8)	0.2179
Hypertension	77.4% (41)	80.9% (38)	0.8067
Recent myocardial infarction	15.1% (8)	6.4% (3)	0.2096
Previous stroke	17% (9)	25.5% (12)	0.3327
Peripheral vascular disease	15.1% (8)	14.9% (7)	1.0000
Atrial fibrillation	43.4% (23)	25.5% (12)	0.0923
Pre-op dialysis	0% (0)	2.1% (1)	0.4700
Previous cardiac surgery	22.6% (12)	19.1% (9)	0.8067
Previous PCI	20.8% (11)	27.7% (13)	0.4855
NYHA I	0% (0)	0% (0)	
NYHA II	5.7% (3)	14.9% (7)	0.1831
NYHA III	71.7% (38)	76.6% (36)	0.6513
NYHA IV	22.6% (12)	8.5% (4)	0.0619
Denied surgical AVR	60.4% (32)	76.6% (36)	0.0912
Pre-op creatinine mmol/L	101.1 (42.2)	109.2 (63.6)	0.4542
EuroSCORE I	25.3 (12.3)	18,2 (10.7)	0.0030
EuroSCORE II	7.8 (4.6)	5.8 (4.8)	0.0378
STS score	7.1 (4.4)	6.3 (7.5)	0.4880
Valve-in-Valve	0% (0)	6.4% (3)	0.1003

Patient characteristics for the patients in the study

For procedural data, the Lotus group had a shorter procedure time (82.9 ± 31.0 vs 118.7 ± 99.7 min, *p* < 0.05), lower frequency of rapid pacing (22% vs 100%, *p* < 0.0001), lower frequency of general anesthesia (39% vs 100%, *p* < 0.0001), less periprocedural bleeding (105 ± 160 vs 203 ± 287 ml, *p* < 0.05, Table 3). Early safety according to the VARC-2-criteria was similar between the groups (Table 4). The Lotus group had a trend towards a higher stroke rate (9% vs 0%, *p* = 0.0585). Of the 5 patients that had a periprocedural stroke, two were major and three were minor with a good recovery.

**Table 3:** Procedural data

	Lotus (*n* = 54)	Sapien (*n* = 47)	*p*-value
Procedural time (min)	82.9 (31.0)	118.7 (99.7)	0.0169
Fluoroscopy time (min)	29.7 (12.3)	26.2 (13.3)	0.1637
Contrast (mL)	92.6 (31.0)	99.3 (32.1)	0.2856
General anesthesia	39.0% (21)	100% (47)	0.0000
Pre-dilatation	59.3% (32)	83% (39)	0.0089
Post-dilatation	0% (0)	27.7% (13)	0.0000
Rapid Pacing	22.2% (12)	100% (47)	0.0000
Per-op bleeding (mL)	105.1 (159.9)	202.8 (287.4)	0.0355
Heart-lung machine (unplanned)	1.9% (1)	8.5% (4)	0.1927
New pacemaker	15.1% (8)	6.8% (3)	0.2172
Aortic valve malpositioning	0% (0)	0% (0)	
Valve migration	0% (0)	0% (0)	
Valve embolization	0% (0)	0% (0)	
Ectopic valve deployment	0% (0)	0% (0)	
TAV-in-TAV deployment	0% (0)	0% (0)	

Procedural data in all 54 Lotus procedures, but new pacemaker reported for the 53 patients

Device success according to the VARC-2-criteria were 98% (all but one patient) for the Lotus group and 91% (all but 4 patients) for the Sapien group (*p* = 0.1809, Table 4). In the Lotus group, in one patient the device malfunctioned when a reposition was performed, and had to be recaptured and removed. The patient had to undergo a reconstruction of the femoral artery as a consequence and received another Lotus valve 42 days later. In the Sapien group, one patient suffered an aortic annular rupture and was converted to open heart surgery but could not be saved, and one patient required a second valve due too low placement resulting in aortic regurgitation. Two *patients in the Sapien group* did not met VARC-2-criteria for valve performance.

**Table 4:** Device Success, Outcome and Safety according to VARC-2

	Lotus (*n* = 53)	Sapien (*n* = 47)	*p*-value
Device Success (n = 54 for Lotus)^a^	98.1% (53)	91.5% (43)	0.1809
Absence of procedural mortality	100% (54)	97.9% (46)	0.4653
Correct positioning of single valve in correct anatomical position	98.1% (53)	97.9% (46)	1.0000
Intended performance of prosthetic heart valve	100% (54)	95.7% (45)	0.2141
Early safety at 30 days	96.2% (51)	89.4% (42)	0.2486
All-cause mortality	3.8% (2)	10.6% (5)	0.2486
All stroke	9.4% (5)	0% (0)	0.0585
Life-threatening bleeding	1.9% (1)	10.6% (5)	0.0965
Acute kidney injury stage I	1.9% (1)	14.9% (7)	0.0244
Acute kidney injury stage II	1.9% (1)	0% (0)	1.0000
Coronary artery obstruction requiring intervention	0% (0)	2.1% (1)	0.4700
Major vascular complication	1.9% (1)	6.4% (3)	0.4968
Valve-related dysfunction requiring repeat procedure	0% (0)	0% (0)	
Clinical efficacy at 30 days			
Mortality	3.8% (2)	10.6% (5)	0.2486
All stroke	9.4% (5)	0% (0)	0.0585
Major stroke	3.8% (2)	0% (0)	0.4968
Rehospitalization for valve-related symptoms	1.9% (1)	6.4% (3)	0.3393
Valve endocarditis	0% (0)	0% (0)	
Valve related dysfunction	0% (0)	4.3% (2)	0.2184

Device Success, Outcome and Safety according to VARC-2. ^a^For Device success all implantations For Lotus (*n* = 54) are reported, but for 30-day outcome all patients are reported (*n* = 53)

**Table 5:** Pre-and post-operative echo

Pre-operative	Lotus (*n* = 53)	Sapien (*n* = 47)	*p*-value
Ejection Fraction			
EF > 50%	67.9% (36)	55.3% (26)	0.2203
EF 30-50%	26.4% (14)	29.8% (14)	0.8241
EF < 30%	5.7% (3)	14.9% (7)	0.1831
Mitral regurgitation			
None	5.7% (3)	4.3% (2)	1.0000
Trace	34% (18)	23.4% (11)	0.3797
Mild	43.4% (23)	48.9% (23)	0.6882
Mild-Moderate	7.5% (4)	17% (8)	0.2179
Moderate	7.5% (4)	6.4% (3)	1.0000
Aortic stenosis			
Peak velocity	4.5 (0.5)	4.2 (0.6)	0.0141
Peak gradient	80 (17.9)	71.7 (18.8)	0.0275
Aortic valve area (cm2)	0.6 (0.2)	0.6 (0.2)	0.0860
Post-operative	Lotus (n = 50)	Sapien (n = 42)	*p*-value
Ejection fraction			
EF > 50%	74% (37)	64.3% (27)	0.2171
EF 30-50%	24% (12)	26.2% (11)	1.0000
EF < 30%	2% (1)	9.5% (4)	0.1841
Aortic regurgitation			
None	58% (29)	26.2% (11)	0.0020
Trace	32% (16)	21.4% (9)	0.2505
Mild	10% (5)	47.6% (20)	0.0002
Mild-moderate	0% (0)	4.8% (2)	0.2184
Moderate	0% (0)	0% (0)	
Mitral regurgitation			
None	4% (2)	0% (0)	0.4982
Trace	36% (18)	47.6% (20)	0.4114
Mild	46% (23)	42.9% (18)	0.4881
Mild-Moderate	0% (0)	4.8% (2)	0.1669
Moderate	14% (7)	4.8% (2)	0.1669
Aortic stenosis			
Peak velocity	2.1 (0.4)	2 (0.5)	0.0838
Peak gradient	18.9 (6.9)	16.5 (8.3)	0.1405

Pre-and post-operative echocardiography re-assessed by one blinded investigator

The re-assessed and blinded post-operative echocardiography showed that the Lotus valve had less paravalvular leakage, 58% had no PVL compared to 26% for Sapien (*p* < 0.005). In the Lotus group 10% had a mild PVL compared to 48% in the Sapien group (*p* < 0.0005). There were no patients with mild-moderate or moderate PVL in the Lotus group compared to 10% in the Sapien group (Table 5).

**Fig. 2:**
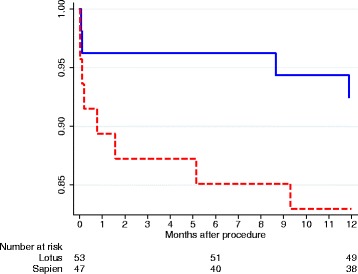
Kaplan-Meier estimated of 1-year survival for Boston Lotus vale (*solid blue line*) and Edwards Sapien valve (*broken red line*)

The thirty day mortality was 3.8% (2 patients) in the Lotus group and 10.6% (5 patients) in the Sapien group (*p* = 0.2486, Table 4). One year mortality was 7.5% (4 patients) in the Lotus group and 17.0% (7 patients) in the Sapien group (*p* = 0.3397, Fig. 2).

## Discussion

The aim of this study was to evaluate whether changing from a first-generation valve to a second-generation valve could be performed safely without affecting outcome for the patients. The study can conclude that safety and efficacy are maintained up to one year.

Despite the differences in the demographics between the groups, it is interesting to compare outcome in terms of prosthesis function, as it determines treatment effect and outcome. Paravalvular leakage is still the Achilles heel of TAVR, and several studies have demonstrated the negative impact of short and long-term mortality from PVL [11–13]. We found a large difference in paravalvular leakage. For the Lotus valve, 90% had none or trace PVL, whereas for the Sapien 48% had none or trace PVL by blinded echo evaluation. There were no mild-moderate or above PVL for the Lotus, whereas the Sapien had 5% mild-moderate PVL. It should be noted that the majority of Sapien cases were performed with Sapien XT, and the newer Sapien-3 has been designed to reduce the frequency of PVL [14, 15]. These figures can still be benchmarked against the incidence of moderate or above PVL in larger studies, where the Lotus valve system had a 1% frequency, the Sapien 3 had a 3,3% frequency, and the Sapien XT had a 13% frequency [7, 14] of moderate to severe PVL. However, the Sapien-3 is not retrievable or repositionable, and PVL can only be addressed with post-dilation. This will likely result in a higher degree of PVL for Sapien-3 compared to Lotus in larger series. The results of 90% with none or trace PVL for the Lotus system is in range, and even better than, the REPRISE II study where 80% came in this category [9]. There were no measurable differences in peak gradient over the valve after implantation between the groups.

Although not statistically significant, the permanent pacemaker rate was numerically higher in the Lotus group as compared to the Sapien group. The Sapien group had 7% pacemaker rate, which is in line with a recent large meta-analysis were the median pacemaker rate was 6% [16]. The Lotus group had a 15% pacemaker rate, which is lower than the 36% reported in the REPRISE I Study and the 29% reported in the REPRISE II study [7, 8]. The best explanation for this is probably that we have adopted a new deployment method for the valve, where we keep the valve in a high position during the entire deployment; never allowing Lotus to drop down into the outflow tract of the ventricle as compared to the traditional way of deploying the valve where retraction is performed in the outflow tract that potentially can scrape the septum and damage the conduction system. Another explanation is a careful pre-operative assessment with gated computer tomography in order to avoid oversizing of the valve. Historically, the high pacemaker rate has been one of the drawbacks with the Lotus valve, but maybe an improved deployment technique as outlined above can address this.

There were no statistical differences in either 30-day or 1-year mortality between the groups, despite Lotus-patients being older with more co-morbidities, had higher pre-gradient and higher EuroScore I and II. This may be attributed to several other factors such as patient selection. It still shows that the Lotus Valve can safely be adopted in a TAVR centre with equally good, and potentially better, outcome. The safety aspect of a second generation valve will be pivotal once intermediate risk patient are considered for a TAVR, as surgery not only places the correct valve in the correct position with minimal PVL, but also has a high predictable safety of the procedure. It will therefore not suffice to only address malpositioning and PVL with a second-generation valve, but safety should never be jeopardized.

There was trend towards a higher stroke rate with the Lotus valve. Two patients experienced a major stroke which was also the cause of death in the Lotus cohort, whereas three had a minor stroke with good recovery. The Lotus system has a larger diameter (18 F and 20 F ID), this may account for this observation, but interestingly French size did not reflect in higher bleeding or vascular complications. The system is more rigid which also could be a reason for the increased stroke rate. We believe this should warrant for care in porcelains aortas or aortas with severe tortuosity, particularly in the learning phase.

The main limitations of this analysis are the relatively small sample size and the lack of randomization between the groups. As the Lotus group was performed later in time, they could benefit from improvements in perioperative care, such as conscious sedation and more frequent use of closure devices and more experienced operators. Moreover there is a selection bias, as patients not suited for a Lotus valve in the later period received a Sapien valve. The reason for this was poor access, too large or too small annulus. On the other hand we preferred the Lotus valve in patients with small sinuses of Valsalva or short distance to the coronaries as it is repositionable. Another aspect is that we have not taken into account the learning curve for the new valve in this study, potentially distorting results in favor of the older valve. Still we believe that the material can be used to test the hypothesis that changing to a new generation valve does not harm the patients, despite the learning curve associated with a new device. One strength of the study is that we had all echocardiographic examinations re-assessed by a blinded, independent and experienced echocardiographer, which underlines our findings regarding the reduced PVL in the Lotus group. In the absence of randomized studies, we believe that this study can be used both for generating hypothesis for future studies, and decisions which valve to use.

## Conclusions

Despite the inherent difficulties in performing a non-randomized retrospective study with subsequent differences between the groups makes it treacherous to draw any far-reaching conclusions. Still, as there were a lower rate of PVL together with a trend towards improved outcome according to VARC-2 criteria and lower 30-day and 1-year mortality, it is reasonable to conclude that patients are not harmed by introduction of repositionable valve in a department.

## Abbreviations

ACT: Activated clotting time
CT: Computed tomography
PVL: Paravalvular leak
TAVR: Transcatheter aortic valve replacements
VARC2: Valve academic research consortium
